# Textural equilibrium melt geometries around tetrakaidecahedral grains

**DOI:** 10.1098/rspa.2017.0639

**Published:** 2018-04-11

**Authors:** John F. Rudge

**Affiliations:** Bullard Laboratories, Department of Earth Sciences, University of Cambridge, Madingley Road, Cambridge CB3 0EZ, UK

**Keywords:** textural equilibrium, permeability, porosity, dihedral angle, partially molten

## Abstract

In textural equilibrium, partially molten materials minimize the total surface energy bound up in grain boundaries and grain–melt interfaces. Here, numerical calculations of such textural equilibrium geometries are presented for a space-filling tessellation of grains with a tetrakaidecahedral (truncated octahedral) unit cell. Two parameters determine the nature of the geometries: the porosity and the dihedral angle. A variety of distinct melt topologies occur for different combinations of these two parameters, and the boundaries between different topologies have been determined. For small dihedral angles, wetting of grain boundaries occurs once the porosity has exceeded 11%. An exhaustive account is given of the main properties of the geometries: their energy, pressure, mean curvature, contiguity and areas on cross sections and faces. Their effective permeabilities have been calculated, and demonstrate a transition between a quadratic variation with porosity at low porosities to a cubic variation at high porosities.

## 1. Introduction

The physical properties of partially molten materials depend crucially on the geometry of melt at the scale of individual grains. Properties like permeability or electrical conductivity can be radically different depending on whether melt forms a connected network or not. The aim of this contribution is to better understand the controls on the geometry of melt networks in order to ultimately better understand the physical properties of partially molten materials.

Surface energy plays a key role in determining melt network geometry. In the absence of external forcing, partially molten materials tend to a state of textural equilibrium in which the surface energies bound up in grain boundaries and grain–melt interfaces are minimized. While in many situations a state of textural equilibrium is not achieved (due to the action of additional mechanical and chemical processes), it provides an important reference model for understanding melt geometry.

It is straightforward to write down a mathematical statement of textural equilibrium, but solving the resulting equations is much less straightforward. Early work [1–4] provided analytical solutions in some simple special cases, or used fairly rough approximations to the geometry. Only in the late 1980s was the simplest three-dimensional problem—four grains meeting at a junction with tetrahedral symmetry—solved fully numerically by von Bargen & Waff [5], Cheadle [6] and Nye [7]. This simple model with tetrahedral symmetry provides important insights into when a melt network is expected to be connected, and also provides constraints on the expected permeability [5,6,8] and electrical conductivity [6,9] of such networks. However, the tetrahedral-symmetry junctions have an important drawback: there is no space-filling solid phase compatible with such junctions. It is thus difficult to use the results from these studies in models which need to describe processes occurring within individual solid grains (e.g. as needed in modelling diffusion creep).

This article provides an exhaustive account of textural equilibrium melt geometries around a particular choice of solid grains which do fill space. In the absence of melt, these chosen grains take the shape of plane-faced tetrakaidecahedrons (truncated octahedrons). The problem has a high degree of symmetry, and is only marginally more complex than the problem tackled by von Bargen & Waff [5], Cheadle [6] and Nye [7]. Indeed, this problem has already been tackled in a series of recent contributions by Ghanbarzadeh *et al.* [10–14], using a novel level-set method that allows for greater flexibility in grain shape than the approach taken here. However, there are some important differences between the results of that study and this study, which will be discussed in detail in §8. The main advantage of the approach taken here over that of Ghanbarzadeh *et al.* [10] is that this study exploits all the symmetries of the problem, which makes it easier to resolve the fine details of the melt geometries.

This article is organized as follows. First the model is described, along with a recap of some well-known mathematical results about textural equilibrium. Accounts are then given of the main properties of the melt topologies: energy, pressure, contiguity, and areas on contacts and cross sections. Permeability calculations are then discussed, and are followed by a more general discussion relating these calculations to previous work. Appendices provide more detail on the numerical methods, and provide some analytical solutions for special cases.

## 2. The model

**Figure 1.**
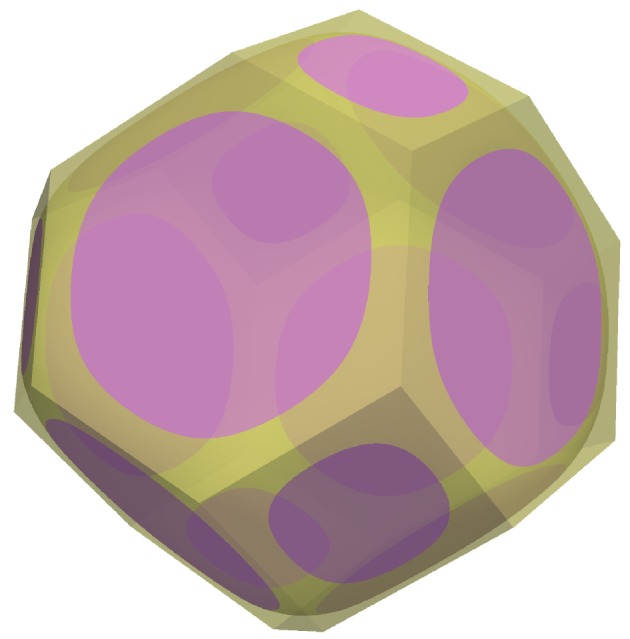
Three-dimensional rendering of the tetrakaidecahedral unit cell. Grain–grain (solid–solid) contacts are shown in pink. The region where melt (liquid) is present is shown in yellow. Translucency has been used to allow a view through the grain.

The model geometry consists of an infinite tessellation of tetrakaidecahedral unit cells as depicted in figure 1. In the absence of melt, a single grain occupies the whole of a unit cell, and the grain takes the shape of a plane-faced tetrakaidecahedron (truncated octahedron). The faces of the tetrakaidecahedron form the grain boundaries (solid–solid contacts). When melt is present, it is assumed that grain boundaries continue to lie along the faces of the tetrakaidecahedral cell, although the area and shape of these contacts are allowed to vary. Textural equilibrium involves the minimization of surface energy *E* [1,4],

2.1 $$E=\frac{1}{2}\gamma_{\mathrm{ss}}A_{\mathrm{ss}}+\gamma_{\mathrm{sl}}A_{\mathrm{sl}},$$

where *γ*_*ss*_ and *γ*_*sl*_ are the surface energies per unit of area of grain–grain (solid–solid) contacts and grain–melt (solid–liquid) contacts, respectively. *A*_*ss*_ and *A*_*sl*_ are the corresponding areas of solid–solid and solid–liquid contacts per unit cell. In this work, it will be assumed that *γ*_*ss*_ and *γ*_*sl*_ are isotropic and constant. The factor of $\frac{1}{2}$ in (2.1) arises from the fact that the grain boundaries are on the faces of the unit cell, so per unit cell each solid–solid contact only counts for half in the total surface energy [4].

**Figure 2.**
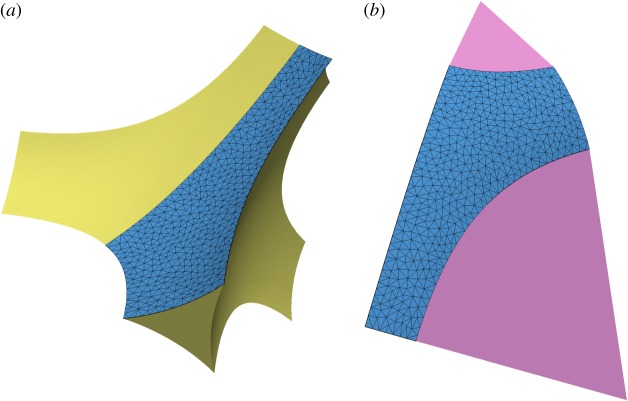
The fundamental computational domain for a porosity *ϕ*=0.03 and dihedral angle *θ*=30^°^. (*a*) Melt quadruple junction showing the solid–liquid interface with the fundamental computational domain in blue with finite-element triangulation in black. The rest of the melt junction can be obtained from the fundamental domain by applying the eight elements of the point group *D*_2*d*_ (tennis-ball symmetry). (*b*) The same fundamental computational domain in blue, but shown in pink are the solid–solid contacts (grain boundaries) associated with the fundamental domain. The full grain can be produced by application of the 48 elements of the point group *O*_*h*_.

Minimization of *E* is performed subject to constraints, which reflect assumptions about the geometry. Here, grain centres are assumed to reside on a body-centred cubic (bcc) lattice, all grain boundaries are assumed to be planar, and all grains are identical. The unit cell shown in figure 1 is the Wigner–Seitz cell of the bcc lattice of grain centres. As a consequence of the cubic symmetry, the basic computational domain can be reduced to a single region that is 1/48th of a grain or 1/8th of a quadruple junction (figure 2).

Grain centres are fixed in these calculations, and thus there is a constant distance *d* between opposing square faces in the unit tetrakaidecahedral cell. The volume of the unit cell is *V*_*cell*_=*d*^3^/2 and it has area $A_{\mathrm{cell}}=\frac{3}{4}(1+2\sqrt{3})d^{2}$. Each edge has length $a=d/(2\sqrt{2})$. The volume fraction of melt is prescribed, by introducing a Lagrange multiplier *λ* and minimizing the functional *J*,

2.2 $$J=E+\lambda (V_{l}-V_{l}^{*}),$$

where *V*_*l*_ is the volume of liquid in the domain and *V* *_l_ is the target (prescribed) value.

The variational problem is discretized by representing the surface by a finite-element mesh of triangles, using the Surface Evolver software [15–17]. Numerical optimization is used to find the unknown mesh node coordinates that minimize the surface energy subject to the given constraints. Further details on the numerical methods can be found in appendix A.

At a minimum, *δJ*=0, and from variational calculus it follows that, at the minimum, *λ*=λ*, where

2.3 $$\lambda^{*}=2\gamma_{\mathrm{sl}}H.$$

Here, *H* is the mean curvature of the solid–liquid surface, defined by

2.4 $$H\equiv \frac{1}{2}\left(\frac{1}{R_{1}}+\frac{1}{R_{2}}\right)\equiv \nabla_{s}\cdot \text{n},$$

where *R*_1_ and *R*_2_ are the principal radii of curvatures, and **n** is the normal vector (chosen here to point outwards from solid/inwards to liquid); ∇_*s*_⋅ represents the surface divergence operator. Thus, in textural equilibrium, the mean curvature of the solid–liquid surface is a constant. Moreover, the Lagrange multiplier enforcing the volume constraint has a physical interpretation: it represents the pressure difference between solid and liquid, λ*=*ΔP*=*P*_*s*_−*P*_*l*_, and (2.3) is the Young–Laplace equation relating pressure differences to mean curvature.

**Figure 3.**
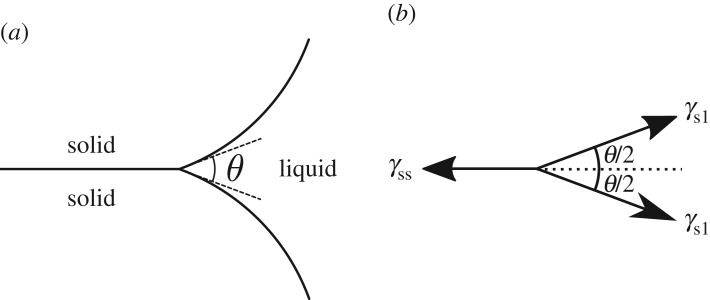
(*a*) The dihedral angle *θ* as the angle at which two solid–liquid surfaces meet a solid–solid surface. (*b*) The corresponding force balance at the triple line.

Also from *δJ*=0 it follows that the following force balance holds along triple lines where three surfaces meet [18]:

2.5 $$\sum_{i=1}^{3}\gamma_{i}\nu_{i}=\text{0},$$

where ***ν***_*i*_ is the co-normal to each surface at the triple line and *γ*_*i*_ is the corresponding surface energy per unit area. This reduces to the familiar expression for the dihedral angle *θ* at which two solid–liquid surfaces meet a solid–solid surface (figure 3),

2.6 $$\cos\frac{\theta }{2}=\frac{\gamma_{\mathrm{ss}}}{2\gamma_{\mathrm{sl}}}.$$

It should be noted that the approach taken here, of discretizing the variational problem (minimize *J* in (2.2)), differs from the approach taken in the classic studies by von Bargen & Waff [5], Cheadle [6] and Nye [7], and also the more recent work by Ghanbarzadeh *et al.* [10]. The starting point for all of these studies is the statement that the solid–liquid surfaces are of constant mean curvature and meet the solid–solid surfaces at the dihedral angle. These studies are all based on a numerical discretization of the mean curvature of the solid–liquid surfaces, whereas here in discretizing *J* only areas and volumes are discretized. The approach taken here, working from the variational problem, is essentially identical to the approach pioneered by Beeré [1], although here a more refined discretization is used.

By scaling, the variational problem can be reduced to being a function of just two dimensionless parameters: the porosity *ϕ* (the volume fraction of melt) and the dihedral angle *θ*. For example, if *E* represents the surface energy contained in one tetrakaidecahedral cell, then a scaled energy can be defined by *E*^′^=*E*/(*γ*_*sl*_*A*_*cell*_), where *A*_*cell*_ is the area of the bounding tetrakaidecahedral cell ($A_{\mathrm{cell}}=\frac{3}{4}(1+2\sqrt{3})d^{2}$). All results in this article are presented using scaled variables as functions of *ϕ* and *θ*.

## 3. Melt topologies

**Figure 4.**
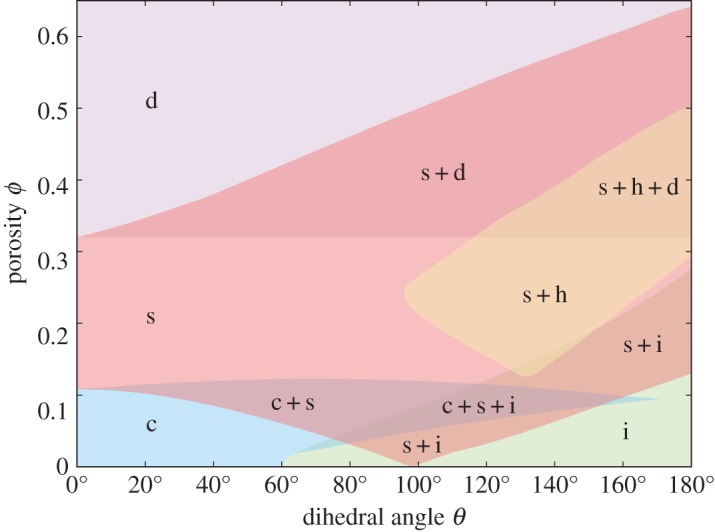
Regime diagram showing regions where different melt topologies have been found to exist. ‘c’ denotes melt connected along grain edges (figure 5). ‘i’ denotes melt in isolated pockets at the grain corners (figure 6). ‘s’ denotes melt that wets the square faces of the grains but not the hexagonal faces (figure 7). ‘h’ denotes melt that wets the hexagonal faces but not the square faces (figure 8). ‘d’ denotes disaggregation, where grains are isolated spheres surrounded by melt. Pluses indicate regions where multiple topologies are found.

One of the key features of textural equilibrium is that different kinds of melt topology are possible for different values of the dihedral angle and at different porosities [2,3]. For example, there is the well-known result that, at small porosities, the melt network is only connected along the grain edges for dihedral angles less than 60^°^. The problem tackled here allows for a rich variety of melt topologies as a function of porosity and dihedral angle, which are summarized in the regime diagram of figure 4 and discussed below. For some combinations of parameters it is possible to find multiple topologies: each is a local minimum of the energy functional, but not necessarily a global minimum.

**Figure 5.**
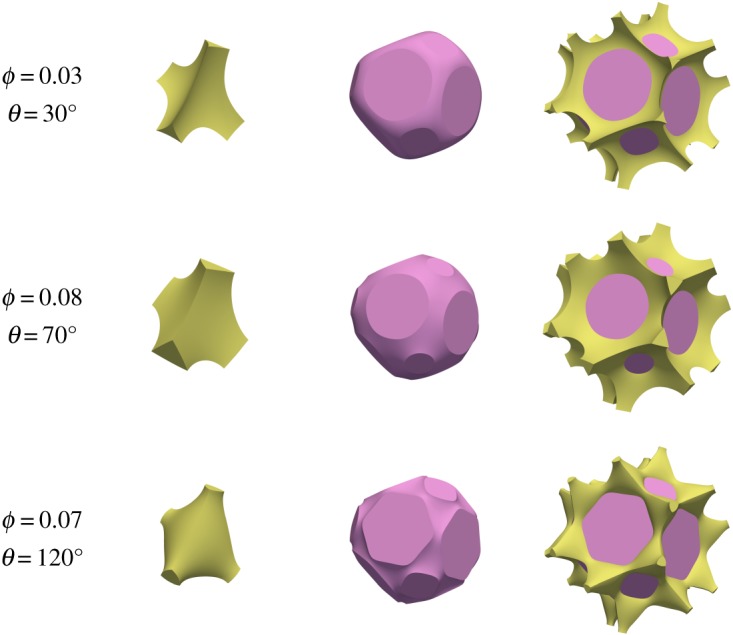
Examples of melt topology ‘c’, connected along grain edges. On the left, in yellow,is a view of the quadruple junction. In the middle, in pink, is a view of an individual grain. On the right is a view of an individual grain + melt. Corresponding labels give the porosity *ϕ* and dihedral angle *θ*. The first two rows have positive mean curvature; the final row has negative mean curvature.

**Figure 6.**
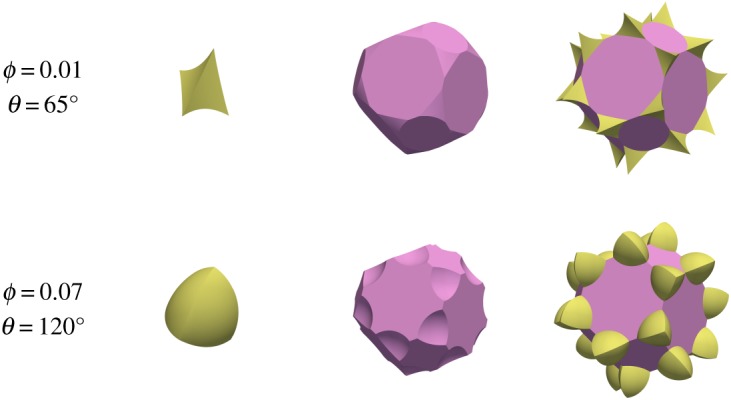
Examples of melt topology ‘i’, where melt is isolated at the grain corners. Note that *θ*=65^°^ has positive mean curvature, whereas *θ*=120^°^ has negative mean curvature. Note that the bottom row has the same combination of porosity and dihedral angle as the bottom row of figure 5.

The first type of topology, marked ‘c’ in figure 4, has melt forming a connected network along the grain edges. Examples of such a topology are shown in figure 5, and are broadly similar to those depicted by von Bargen & Waff [5], Cheadle [6] and Nye [7], except that the melt junction does not have tetrahedral symmetry. The second type of topology, ‘i’ (figure 6), which consists of melt isolated at grain corners, is also broadly similar to previous calculations which assumed tetrahedral symmetry, for which analytical expressions are available [2]. At very large porosities the grain–grain contacts disappear and grains are completely surrounded by melt. This is marked as ‘d’ for disaggregated in figure 4, where the minimum energy configuration simply consists of spherical grains. ‘d’ topologies exist for all porosities greater than $1-\pi \sqrt{3}/8\approx 32\%$.

**Figure 7.**
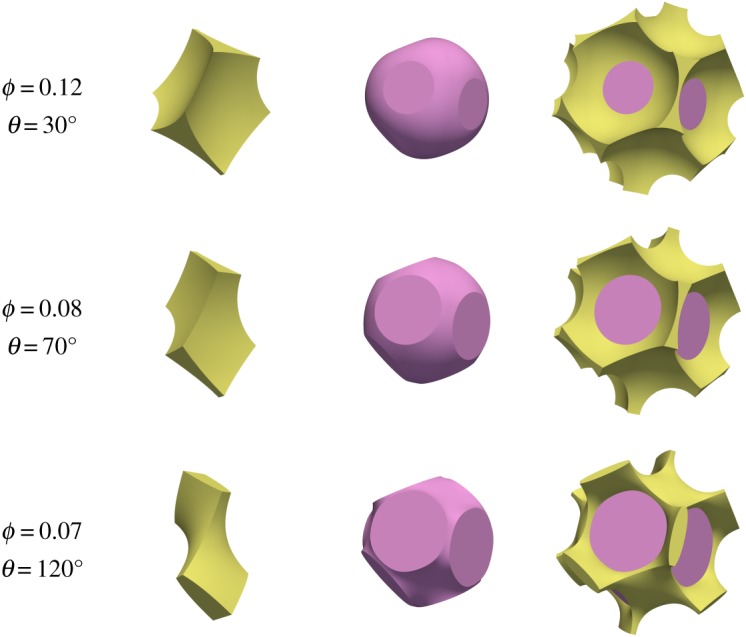
Examples of melt topology ‘s’, where melt is connected and wets the square faces. Note that the bottom two rows have the same combinations of porosity and dihedral angle as the bottom two rows of figure 5.

**Figure 8.**
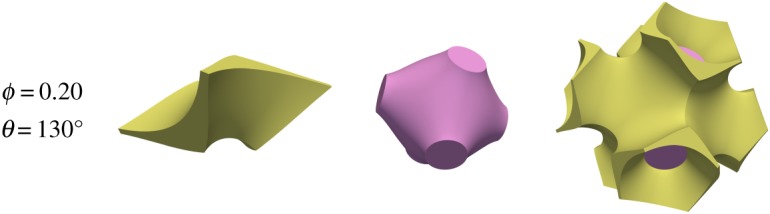
An example of melt topology ‘h’, where melt is connected and wets the hexagonal faces.

The problem considered here admits additional melt topologies because of the lower degree of symmetry of the quadruple junction. These are depicted in figures 7 and 8. The first of these represents the case ‘s’, where the square faces become wetted but the hexagonal faces do not. The second represents the case ‘h’, where the hexagonal faces are wetted but the square faces are not.

For low porosities, the dihedral angles for which topologies ‘c’ and ‘i’ are found are essentially the same as found for the problems with tetrahedral symmetry: isolated solutions exist only for dihedral angles greater than 60^°^. For dihedral angles greater than this, there is a region of overlap between the ‘c’ and ‘i’ topologies as porosity increases (the region between the ‘pinch-off’ and ‘wetting’ boundaries as described by von Bargen & Waff [5]).

Perhaps the most important new transition in the present study is the transition from ‘c’ to ‘s’ topologies as porosity increases (i.e. the preferential wetting of the smaller square faces). For low dihedral angles, this occurs close to *ϕ*=0.11, but for larger dihedral angles there is an overlap where both ‘c’ and ‘s’ topologies exist. The wetting of square faces at around *ϕ*=0.11 is also seen in calculations of wet Kelvin foams [19–24]. Wet Kelvin foams are essentially a limiting case of the problem considered here: the case where the dihedral angle approaches 0^°^. The network of melt considered here is termed a Plateau border network in the foam literature. The only difference is that a wet Kelvin foam allows for some curvature along what are the grain–grain contacts in the present model, but such curvature is extremely slight, and unlikely to significantly affect where the transition to wetted square faces occurs.

One curious feature of figure 4 is that for dihedral angles just below 100^°^ the ‘s’ solutions exist down to very small porosities. If such a solution were realizable, there would be the potential for percolation of melt at small porosities even for some dihedral angles greater than 60^°^. However, as will be seen in the next section, such ‘s’ topologies have higher energy than the ‘i’ topologies and are thus less likely to be realized.

It should be noted that figure 4 does not provide a map of all possible melt topologies, only a key subset that have been chosen to be investigated. Firstly, the topologies investigated are only those consistent with the symmetries imposed. Topologies with lower degrees of symmetry are possible, e.g. isolated topologies with melt at some quadruple junctions but not others. Moreover, there are additional isolated topologies possible that are consistent with the imposed symmetry but that have not been calculated here. For example, melt can also be isolated in the middle of the grain edges, or at the centres of the grain faces. One can also have some combination of isolated melt in all three places—edges, faces and vertices of the unit cell. However, at the onset of melting it is expected that melt first forms at the quadruple junctions, so it is the case of melt at these junctions that is typically of most interest.

## 4. Energy and pressure

**Figure 9.**
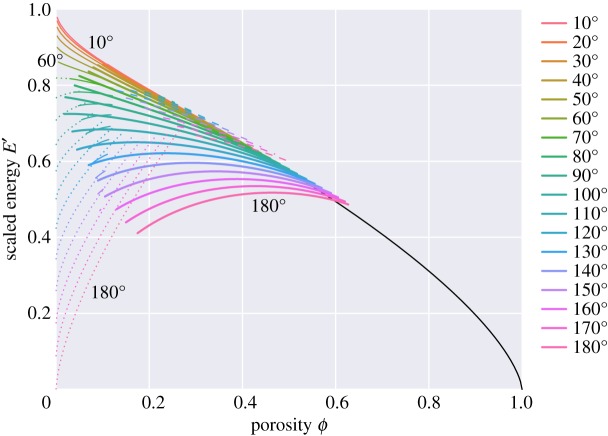
Scaled (dimensionless) energy, *E*^′^=*E*/(*γ*_*sl*_*A*_*cell*_), plotted as a function of porosity *ϕ* for different values of the dihedral angle *θ* (key on the right). In this plot, and subsequent plots, thin solid lines represent connected topology ‘c’, thick solid lines represent square-wetted topology ‘s’, dotted lines represent isolated topology ‘i’ and dashed lines represent hex-wetted topology ‘h’. The black solid line represents disaggregated topology ‘d’ (here independent of the dihedral angle).

Figure 9 plots a scaled surface energy for each of the melt topologies as a function of porosity and dihedral angle. For those regions of *ϕ*−*θ* space that admit multiple topologies, figure 9 can be used to identify which topology has the overall lowest energy (and hence is more likely to be found). For example, the ‘h’ topologies (which wet the hexagonal faces) always have higher energy than the ‘s’ topologies (which wet the square faces). This is what one might intuitively expect—it is more likely for smaller faces to become wetted than larger faces.

The situation is more complicated in the region of overlap between ‘c’ and ‘s’. For small dihedral angles, the ‘c’ topologies have lower energy than the ‘s’ topologies for most of the region of overlap, until close to the boundary where only ‘s’ exists. For larger dihedral angles the ‘s’ topologies are lower energy for most of the range of overlap. Similarly, in the region of overlap between ‘s’ and ‘i’, for lower dihedral angles the ‘i’ topologies are lower energy for much of the region of overlap, whereas at higher dihedral angles the ‘s’ topologies are lower energy for the whole region of overlap.

**Figure 10.**
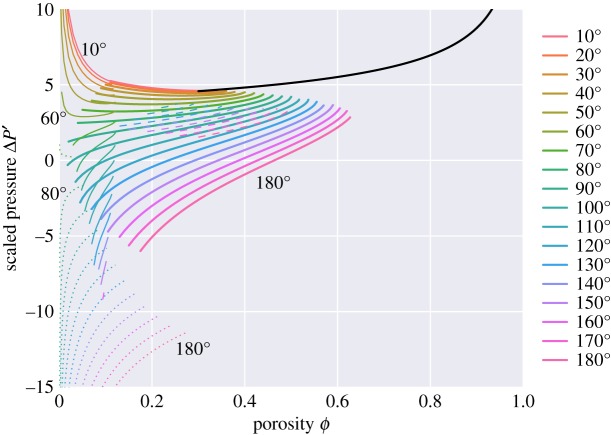
Scaled pressure (or scaled mean curvature), *ΔP*^′^=*ΔPd*/*γ*_*sl*_=2*Hd*, as a function of porosity.

Figure 10 plots a scaled version of the pressure difference *ΔP* between solid and liquid for the various melt topologies. Owing to the Laplace–Young equation (2.3), this is also a plot of a scaled mean curvature. This pressure difference is calculated during the solution of the variational problem as *ΔP*=λ* is the Lagrange multiplier that enforces the fixed volume constraint. The curves in figure 10 are related to the slopes of the curves for energy in figure 9, because of the relationship

4.1 $$\Delta P=-{\frac{\partial E^{*}}{\partial V_{l}^{*}}|}_{V_{\mathrm{cell}}^{*}},$$

which is a consequence of the variational problem. Here, E* represents the energy at equilibrium, and *V* *_l_ and *V* *_cell_ the volumes of the liquid and unit cell, respectively. In the scaled (dimensionless) variables used in figures 9 and 10, this relationship becomes

4.2 $$\Delta P^{\prime }=-\frac{3}{2}(1+2\sqrt{3})\frac{\partial E^{\prime }}{\partial \phi }.$$

Figure 10 shows a number of features that are consistent with previous studies. For example, for the isolated topologies ‘i’, the curvature is positive for *θ*<71^°^ and negative for *θ*>71^°^ [2]. Interestingly, topologies ‘i’, ‘c’ and ‘s’ have zero mean curvature for some combinations of porosity and dihedral angle, and are thus examples of ‘minimal surfaces’ (surfaces defined as having zero mean curvature).

The pressure difference between the two phases becomes singular as the porosity approaches zero, but the form of singularity varies with the dihedral angle. For isolated topologies the porosity dependence is of the form *ΔP*∝*ϕ*^−1/3^ (see (B.5) in appendix B). For small dihedral angles, the melt geometry can be closely approximated by tubes along the grain edges, for which *ΔP*∝*ϕ*^−1/2^ (see (B.16) in appendix B).

## 5. Effective pressure

The pressure plotted in figure 10 is a measure of the change in energy of the system with liquid content, assuming the cell size remains fixed (and thus as the volume of liquid increases, the volume of solid must decrease). As pointed out by Park & Yoon [4], in several physical situations what one would like to know is the change in energy with liquid content, but keeping the solid volume (*V* *_s_) fixed. For example, in problems of compaction or sintering one may have a partially molten medium surrounded by a reservoir of liquid, and want to know whether liquid will be drawn in or expelled from the partially molten medium. Park & Yoon [4] quantify this in terms of an ‘effective pressure’, *P*_*e*_, defined by

5.1 $$P_{e}=-{\frac{\partial E^{*}}{\partial V_{l}^{*}}|}_{V_{s}^{*}}.$$

**Figure 11.**
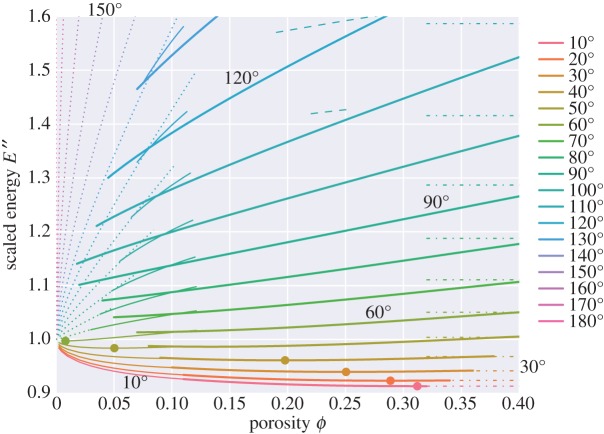
Scaled energy, scaled in the same way as figure 2 of Park & Yoon [4], $E^{\prime \prime }=E/(\frac{1}{2}\gamma_{\mathrm{ss}}A_{\mathrm{cell}0})$. Filled circles show the minimum values, which correspond to zero crossings in figure 12. Dash-dotted lines show the energy for the disaggregated topology ‘d’ (which varies with dihedral angle in this scaling, unlike the scaling in figure 9).

**Figure 12.**
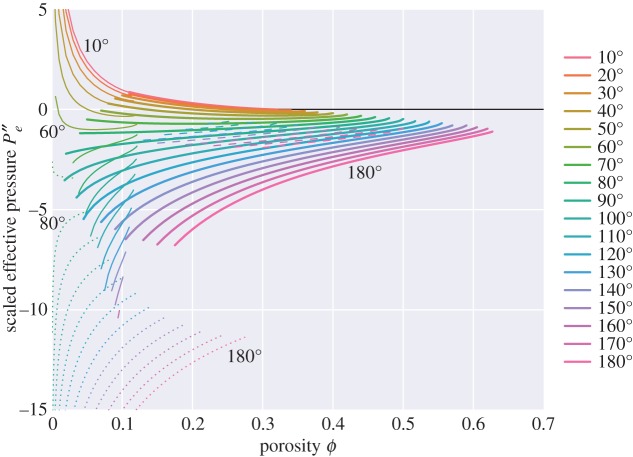
Scaled effective pressure, scaled in the same way as figure 4 of Park & Yoon [4], $P_{e}^{\prime \prime }=P_{e}d_{0}/\gamma_{\mathrm{sl}}$.

Figures 11 and 12 show scaled energy and scaled effective pressure, analagous to figs 2 and 4 of Park & Yoon [4]. They are scaled such that, as porosity varies, the solid volume remains fixed (rather than the cell size). Scaling is made with respect to the length *d*_0_,

5.2 $$d_{0}=d{\left(1-\phi \right)}^{1/3},$$

which is defined as the distance between grain centres in the absence of melt with the same volume of solid grain. The area of the unit cell in the absence of melt is given as $A_{\mathrm{cell}0}=\frac{3}{4}(1+2\sqrt{3})d_{0}^{2}$, and the volume as $V_{\mathrm{cell}0}=d_{0}^{3}/2$. The plot of effective pressure in figure 12 is related to the slope of figure 11 by

5.3 $$P_{e}^{\prime \prime }=-\frac{3}{2}(1+2\sqrt{3}){\left(1-\phi \right)}^{2}\frac{\partial E^{\prime \prime }}{\partial \phi }\cos\frac{\theta }{2}.$$

If the sign of *P*_*e*_ is positive, it indicates that there is a driving force sucking liquid into the partially molten material. If negative, the driving force acts to expel liquid. Zero *P*_*e*_ represents a stable state (a minimum of the scaled energy plotted in figure 11). The key result of figures 11 and 12 is that topologies for dihedral angles greater than 60^°^ are always unstable, and the driving force is such that the two phases try to separate (*P*_*e*_ is always negative). For dihedral angles less than 60^°^ there exists a critical porosity at which the effective pressure is zero, and hence the topology is stable. The smaller the dihedral angle, the larger the critical porosity. If the partially molten medium has a porosity less than this critical porosity there will be a tendency of melt to be drawn in, whereas if it is above this critical porosity there will be a tendency for melt to be expelled.

In Park & Yoon’s original study, solutions with zero effective pressure could be found for dihedral angles up to 75^°^, rather than the 60^°^ limit found here. One difference between Park & Yoon’s study and the present study is that Park & Yoon [4] consider a unit cell taking the shape of a rhombic dodecahedron. However, the discrepancy with the results of this study is likely to have arisen from Park & Yoon’s approximation of the grain–melt surfaces by circular arcs, which is not consistent with the surfaces being of constant mean curvature.

## 6. Geometrical properties

**Figure 13.**
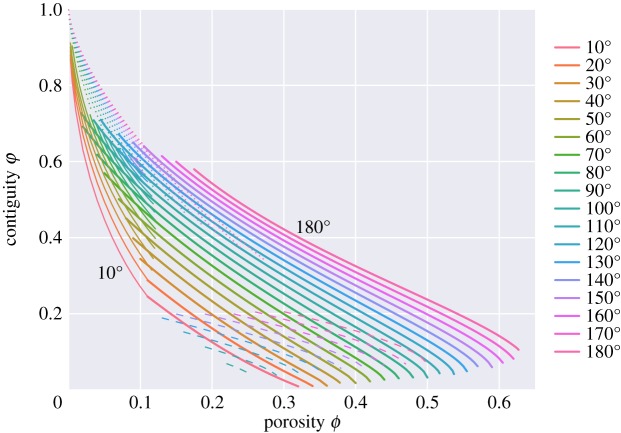
Contiguity. *φ*=*A*_*ss*_/(*A*_*ss*_+*A*_*sl*_).

**Figure 14.**
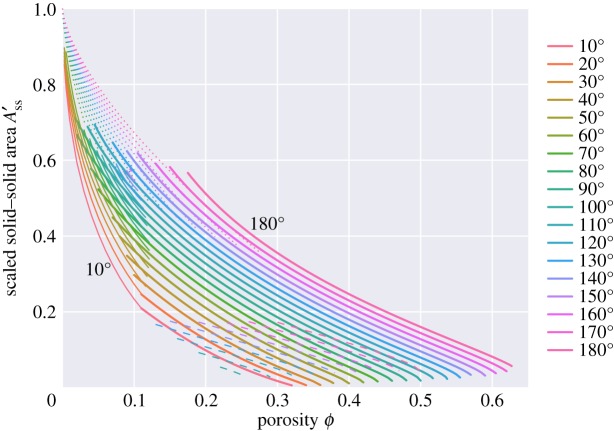
Total area of solid–solid contact, normalized to the area of the unit cell, *A*_*ss*_/*A*_*cell*_.

**Figure 15.**
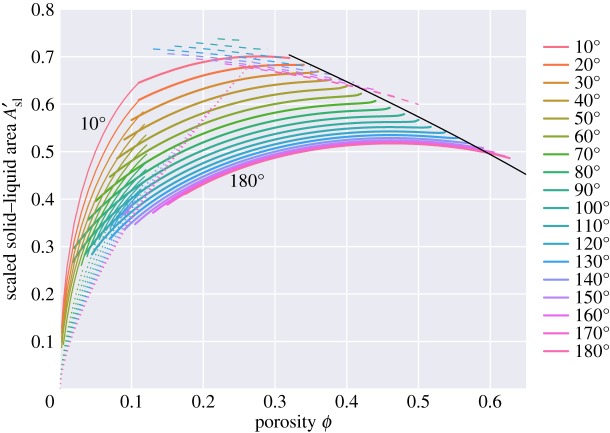
Total area of solid–liquid contact, normalized to the area of the unit cell, *A*_*sl*_/*A*_*cell*_.

**Figure 16.**
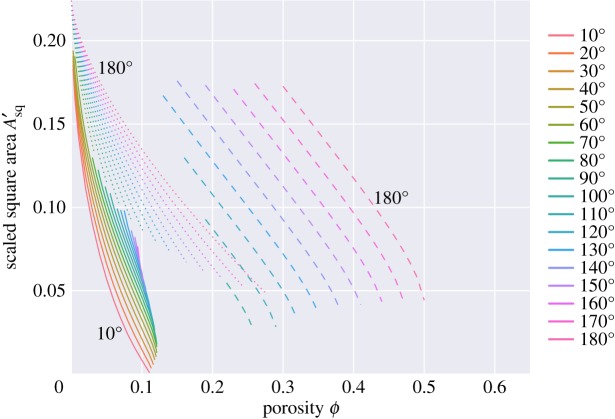
Square solid–solid contact area, normalized to the area of the unit cell, *A*_*sq*_/*A*_*cell*_. The ‘s’ and ‘d’ topologies have zero square contact area and are not shown.

**Figure 17.**
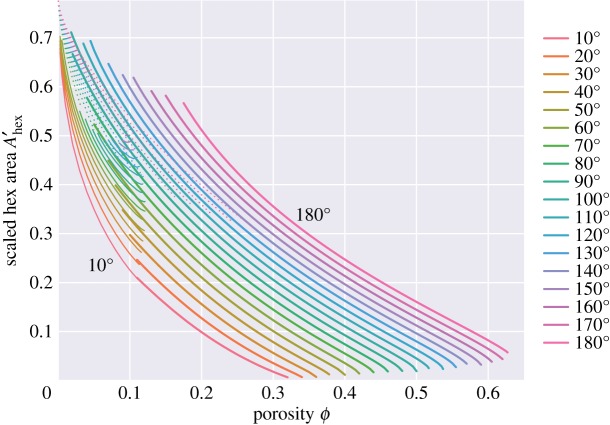
Hexagonal solid–solid contact area, normalized to the area of the unit cell, *A*_*hex*_/*A*_*cell*_. The ‘h’ and ‘d’ topologies have zero square contact area and are not shown.

**Figure 18.**
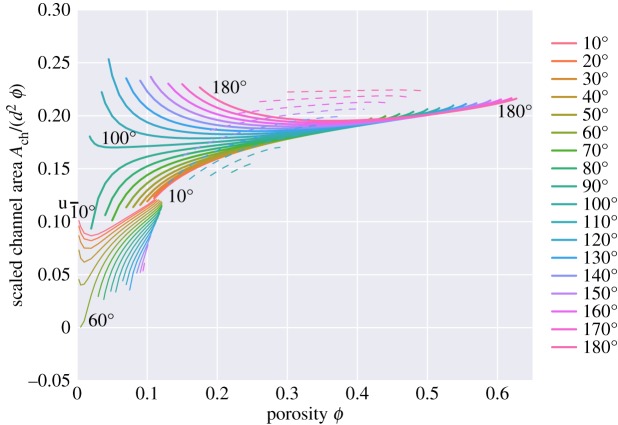
Scaled cross-sectional channel area (sectioned at the mid-grain edge), scaled as *A*_*ch*_/(*d*^2^*ϕ*). Isolated topologies ‘i’ have no melt at mid-grain edges and thus have zero area and are not shown. The black line labelled ‘u’ on the *y*-axis shows the expected channel area for melt tubes of uniform cross section along the grain edges.

Figures 13–18 summarize a number of key geometrical properties. The first of these (figure 13) shows contiguity *φ*,

6.1 $$\varphi =\frac{A_{\mathrm{ss}}}{A_{\mathrm{ss}}+A_{\mathrm{sl}}},$$

which measures the relative area of grain–grain contact to total grain area. Figures 14 and 15 show the individual areas of solid–solid and solid–liquid contact, normalized to the area of the unit cell. Contiguity is used as a fundamental variable in a number of micromechanical models, e.g. in determining elastic properties [25] and effective viscosities due to creep [26]. For small porosities, figure 13 shows the same main trends as observed by von Bargen & Waff [5] and Cheadle [6], with contiguity being larger for larger dihedral angles. Contiguity shows a steady decrease with increasing porosity as more of the grain becomes wetted. There is a notable change in slope during the transition from ‘c’ to ‘s’ topologies, with a shallower slope for the ‘s’ topologies than the ‘c’ topologies.

In this problem, there are two different kinds of grain–grain contact—those associated with the square faces and those associated with the hexagonal faces. Figure 16 plots the areas of the square faces, and figure 17 that of the hexagonal faces. For the ‘c’ topologies, the areas of the square faces decrease as porosity increases, but note that they do not vanish at the boundary between the ‘c+s’ and ‘s’ topologies: thus wetting of the square faces occurs discontinuously from a finite area of square grain–grain contact to a zero area as porosity increases. Only for a zero dihedral angle does the ‘c’ topology smoothly go to zero area of the square face as porosity increases. Such discontinuous jumps in behaviour are common in area minimization problems. The classic example of this is in the minimal surface of revolution problem: finding the soap film which minimizes the area between two parallel circular hoops [27]. Beyond a critical separation, the catenoid solution which joins the two hoops breaks down to the Goldschmidt discontinuous solution with two separate films spanning the two circles. For small dihedral angles, there is a close agreement between these results and those for wet Kelvin foams: hexagonal and square areas reported in figure 5 of [22] are very similar to what one would expect on extrapolating the results of figures 16 and 17 to zero dihedral angle.

Figure 18 plots a scaled version of the channel area *A*_*ch*_ along the middle of each channel edge (i.e. the area of the truncated faces of the quadruple junctions shown in figures 5–8). If the melt resided in tubes along the grain edges of uniform cross section, one would expect $A_{\mathrm{ch}}\approx \sqrt{2}/12d^{2}\phi \approx 0.117851d^{2}\phi$ for small porosities (black line on the *y*-axis in figure 18). For small porosities, and small dihedral angles, figure 18 shows that this value is approached, reflecting the fact that for such porosities and dihedral angles the melt geometry is well approximated by tubes. At small porosities, the mid-edge channel area decreases with increasing dihedral angle, reflecting the fact that more melt resides near the grain vertices than resides near the grain edges. The opposite trend is seen for the ‘s’ topologies at large porosity, where the larger dihedral angles have larger channel areas.

## 7. Permeability

**Figure 19.**
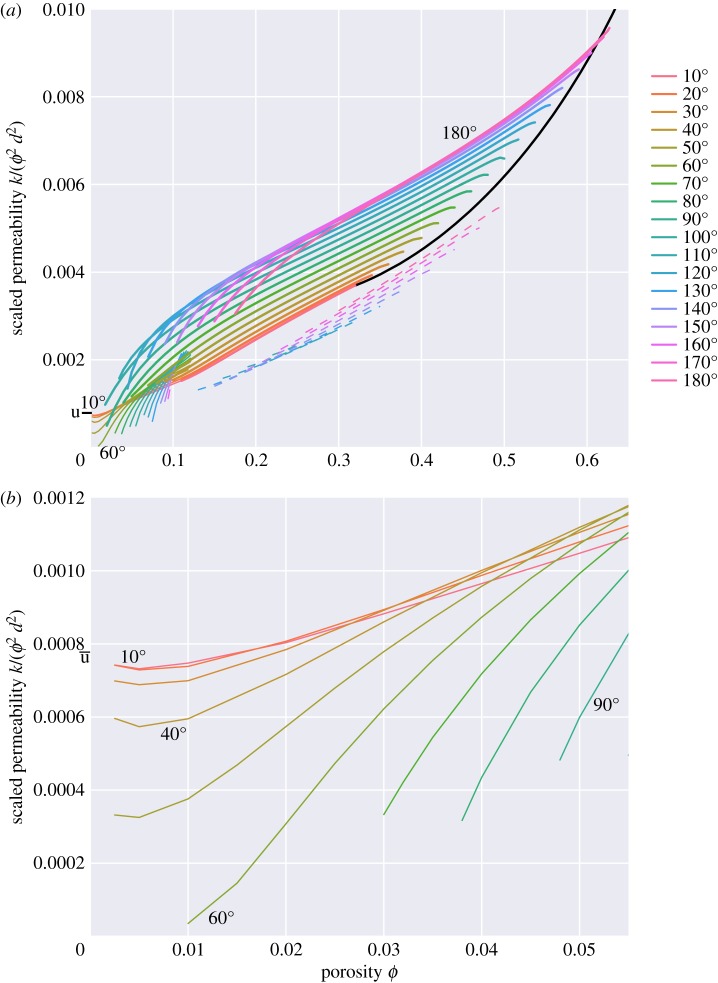
(*a*) Scaled permeability *k*/(*d*^2^*ϕ*^2^). The solid black line in the plot is for topology ‘d’, a bcc array of isolated spheres (the permeabilities calculated in this case are in agreement with previous calculations, e.g. ch 9 of [28]). The short black line labelled ‘u’ on the *y*-axis shows the expected permeability for melt tubes of uniform cross section along the grain edges. (*b*) Same data as shown in panel *a*, but zoomed-in to show the behaviour of ‘c’ topologies at low porosities (similar to figure 14 of von Bargen & Waff [5]).

The effective permeabilities of the melt topologies are shown in figure 19, and details of their calculation can be found in appendix C. Permeabilities are scaled in figure 19 by *d*^2^*ϕ*^2^, based on the expected behaviour for low porosities (see below). Most of the main features of figure 19 can be understood in the context of simpler models. For small porosities, and small dihedral angles, the melt geometry for topology ‘c’ takes the form of tubes along the grain edges. A very simple model of permeability was derived by Frank [29], consisting of tubes of melt with circular cross section lying on the edges of a tetrakaidecahedron. For this model, the permeability is

7.1 $$k_{\mathrm{Frank}}=\frac{\phi^{2}d^{2}}{144\pi \sqrt{2}}\approx 0.00156\phi^{2}d^{2}.$$

As discussed by von Bargen & Waff [5], this formula gives an overestimate of the permeability at low porosity for the melt networks given here, because the melt tubes do not have circular cross section. For a cross-sectional shape more appropriate for low dihedral angles (three circular arcs meeting at zero dihedral angle), the melt flux for a given cross-sectional area and pressure gradient is a factor of 0.505 lower. A modified version of Frank’s formula is then

7.2 $$k_{\text{tubes},\theta \approx 0^{\circ }}\approx 0.000789\phi^{2}d^{2}=\frac{\phi^{2}d^{2}}{1267}$$

and this formula is marked as ‘u’ on the *y*-axis of figure 19. The equation above explains the limiting behaviour seen in figure 19 for low porosities and low dihedral angles, which tend to finite values of *k*/(*ϕ*^2^*d*^2^) as ϕ→0, and whose geometries can be well described by tubes along the grain edges with approximately uniform cross section. For larger dihedral angles the cross sections become much less uniform, with more melt residing at the vertices of grains rather than the edges. The effect of this is to reduce the permeability. von Bargen & Waff [5] provide an approximate formula,

7.3 $$k_{\text{vBW}}\approx 0.000625\phi^{2}d^{2}=\frac{\phi^{2}d^{2}}{1600},$$

which is in good agreement with the results here for small porosities and dihedral angles around 40^°^. Such a formula gives the permeability to within 15% for all porosities less than 2%. The expression quoted by Cheadle [6] (*k*=*ϕ*^2^*d*^2^/3000) seems to underestimate the permeability, and the permeabilities calculated here are much closer to the results of von Bargen & Waff [5].

For larger porosities (greater than 10%), lines on figure 19 are roughly linear, which reflects the fact that at large porosities permeability scales as *k*∝*d*^2^*ϕ*^3^. The *ϕ*^3^ behaviour can also be understood from simpler models, and is the expected behaviour for melt lying as thin sheets along the grain boundaries. A reasonable approximation for 0.1<*ϕ*<0.3 and the ‘s’ topologies is

7.4 $$k_{\text{large }\phi }\approx \frac{\phi^{3}d^{2}}{75},$$

which lies within about 25% of the calculated values for dihedral angles *θ*<60^°^. Indeed, it should be noted that the relative range in permeability is very small when *θ* varies for porosities above 10%: a factor of 2 at most. It is interesting to note that the behaviour of permeability with dihedral angle differs at higher porosities: for high porosity, the larger the dihedral angle, the greater is the permeability; although the magnitude of this effect is rather modest.

Permeability data are often parameterized with a single law of the form *k*=*ϕ*^*n*^*d*^2^/*C* (e.g. [30,31]). Note that such a parametrization in terms of a single *C* and *n* value would not be able to describe all the subtleties seen in figure 19, and particularly the shift from *k*∝*d*^2^*ϕ*^2^ at low porosities to *k*∝*d*^2^*ϕ*^3^ at larger porosities.

## 8. Discussion

As remarked in the Introduction, the textural equilibrium geometries calculated here have also been calculated by Ghanbarzadeh *et al.* [10–14], although these authors consider a more limited range of porosities and dihedral angles. Ghanbarzadeh *et al.* [11] introduce a novel approach to calculating textural equilibrium geometries based on the level-set method. In this approach, the interface between the solid and liquid is represented by the level set of a function, and the interface evolves at a velocity proportional to the surface Laplacian of mean curvature. Over time, the surface should approach a state of constant mean curvature. A partial differential equation determines the evolution of the level-set function, and additional terms are added to the PDE to enforce the dihedral angle constraint. Ghanbarzadeh *et al.* [11] discretize the PDE using high-order finite differences.

One of the advantages of Ghanbarzadeh *et al.*’s approach is that it allows a great deal of geometric flexibility, including the ability to solve for many grains at once with different grain shapes. As such, they do not explicitly impose symmetry on the solution (as is done here). Their method is considerably more computationally expensive than the approach taken here (which can solve for the melt geometries in seconds).

In broad terms, many of the topologies calculated by Ghanbarzadeh *et al.* [11] are similar to those calculated here, but there are some notable discrepancies. One of the most important discrepancies concerns the wetting of the square faces. Ghanbarzadeh *et al.* [10] state that ‘In contrast with prevailing assumptions, the smaller square grain boundaries become fully wetted at *θ*=10^°^ and *ϕ*≥5%.’ As can be seen in figure 4, this study places the transition to wetted square faces at closer to *ϕ*=11% for *θ*=10^°^: a factor of 2 difference. As remarked earlier, transitioning at *ϕ*=11% is consistent with results for wet Kelvin foams [19,22,23].

Another discrepancy is in the calculated mean curvatures of the melt topologies. Hesse *et al.* [32] presented a plot of mean curvature against porosity for isolated ‘i’ and connected ‘c’ topologies for a dihedral angle *θ*=90^°^. In theory, this plot should be similar to the curvature information shown in figure 10, but in fact bears little resemblance. First, Hesse *et al.* [32] showed ‘c’ topologies for porosities as low as 1.5%, whereas the suggested lower bound for ‘c’ topologies here is around 4% (very similar to the pinch-off boundary in fig. 7 of von Bargen & Waff [5]). Moreover, the behaviour of the mean curvature with porosity for the isolated ‘i’ topologies shown by Hesse *et al.* [32] is very different from that seen here. The shape of the isolated topologies is independent of porosity, apart from a scaling of the coordinates [2]. Thus mean curvature *H* should be proportional to *ϕ*^−1/3^ for the ‘i’ topologies, and be singular at zero porosity (as indeed seen in figure 10). The curve shown by Hesse *et al.* [32] does not show this behaviour, and instead appears to curve the wrong way, and seems to approach a finite value for small porosity.

These discrepancies matter, because they lead to different predictions about upscaled physical properties like permeability. Indeed, the permeabilities that have been reported by Ghanbarzadeh *et al.* [13,14] differ from those calculated here by a significant margin, in some cases by almost an order of magnitude. For example, for a dihedral angle of 10^°^, Ghanbarzadeh *et al.* [13,14] report that their permeability data can be fitted well by an expression of the form *k*=*d*^2^*ϕ*^2.6^/595.66. For a porosity of *ϕ*=0.01 this implies *k*/(*d*^2^*ϕ*^2^)≈1×10^−4^, whereas here a value of *k*/(*d*^2^*ϕ*^2^)≈7×10^−4^ has been estimated (figure 19). Thus, Ghanbarzadeh *et al.* [13,14] seem to underestimate the permeability by a factor of 7 for these parameter values. For a porosity of *ϕ*=0.1, Ghanbarzadeh *et al.*’s expression implies *k*/(*d*^2^*ϕ*^2^)≈4×10^−4^, whereas here *k*/(*d*^2^*ϕ*^2^)≈1.4×10^−3^ has been estimated, more than a factor of 3 greater. Perhaps the most probable reason for the discrepancies is that the simulations of Ghanbarzadeh *et al.* [11] are under-resolved, and struggle to accurately capture small-scale variations in the geometry, e.g. with cusps at a small dihedral angle or small radii of curvature at low porosities. The accuracy of the solutions obtained in this study is discussed in appendix A.

Another point of difference between this study and that of Ghanbarzadeh *et al.* is that Ghanbarzadeh *et al.*’s study essentially aims to produce surfaces of constant mean curvature compatible with the dihedral angle constraints. Constant mean curvature is a necessary, but not a sufficient, condition for minimum energy. Constant mean curvature guarantees that the energy is extremized, but does not say whether the surface is a minimum, maximum or a saddle point of the energy. Indeed, it is well known that some solutions of the Euler–Lagrange equations for area minimization problems can be unstable, as in the minimal surface area of revolution problem mentioned earlier. Hence it is possible that the scheme proposed by Ghanbarzadeh *et al.* produces melt topologies that are not minimum energy, and hence would not be found by the approach taken here.

The existence of multiple melt topologies has important consequences for upscaled physical properties (e.g. permeability, electrical conductivity, effective viscosities). First, transitions in such properties can be discontinuous on varying parameters, as the underlying transitions in topology are discontinuous. Moreover, as remarked by von Bargen & Waff [5], and more recently by Hesse and co-workers [14,32], there is the potential for hysteresis in topology and hence hysteresis in upscaled physical properties. For example, suppose the dihedral angle is 40^°^ and porosity is increased slowly from zero such that textural equilibrium is maintained (figure 4). The topology would remain as type ‘c’ until a porosity of 12% when a discontinuous transition to the type ‘s’ topology would occur (wetting of the square faces), when the ‘c’ state is no longer stable. If porosity were then slowly reduced, the topology would stay as type ‘s’ until a porosity of 9% when a discontinuous transition back to ‘c’ would occur, as the grain–melt surfaces begin to touch on the square faces (de-wetting of the square faces). Similar hysteresis can occur in other parts of the regime diagram, for example the ‘i’ to ‘c’ transition discussed by von Bargen & Waff [5].

There are several natural ways in which the present work can be extended. In the same way that this study presents a model with a lower degree of symmetry than the studies by von Bargen & Waff [5], Cheadle [6] and Nye [7], it would be fruitful to examine the consequences of relaxing more of the symmetry assumptions. Symmetry plays a crucial role in constraining the solutions produced here, and topologies which are stable under the symmetry constraints imposed here may not be stable if some of these symmetry constraints are relaxed.

In this study, a tetrakaidecahedral unit cell was chosen, but it would be straightforward to repeat the same calculations for other choices of unit cell, such as the rhombic dodecahedral unit cell used by Park & Yoon [4]. In a tessellation of tetrakaidecahedrons, at each vertex four edges meet, and each vertex is thus said to have a coordination number of 4. In a tessellation of rhombic dodecahedrons, half of the vertices have a coordination number of 4, and half have a coordination number of 6. X-ray tomographic imaging suggests that the predominant coordination number in olivine–basalt systems is 4 [33], so the tetrakaidecahedral unit cell is the more appropriate geometry for the partially molten rocks of the Earth’s mantle.

To simplify the calculations, grain boundaries were forced to be planar, and forced to lie on certain symmetry planes. Grain boundaries are in general not perfectly planar, and these constraints could be relaxed in future work. Indeed it is the curvature of grain boundaries that allows for grain growth and coarsening, an effect that is suppressed in the present calculations by the assumptions that have been made. Another natural extension is to consider the anisotropy in surface energy, both between grains and between the grains and the melt. Anisotropy can lead to faceted solid–liquid and solid–solid interfaces (e.g. [34]) that will present challenges for numerical simulation. Another challenge for the future is to move beyond textural equilibrium, to study the interplay between deformation and melt network geometry.

## 9. Conclusion

The aim of this study has been to produce a simple reference model which describes the geometry of melt within a partially molten material. This model, based around a tessellation of tetrakaidecahedral unit cells, has an advantage over most previous models in that the melt geometry is compatible with a space-filling tessellation of grains. A lot of the fundamental behaviour of the melt topologies calculated here agrees with the classic models of von Bargen & Waff [5], Cheadle [6] and Nye [7], particularly at small porosities. The main differences occur at higher porosities, where topologies that wet the square faces of the tetrakaidecahedral grains exist.

These geometries form a starting point for the calculation of upscaled physical properties which depend on the geometry of melt at the grain scale. One example of such a property has been presented here, namely the permeability, but the intent is to describe a wider range of physical properties in future articles. In particular, calculations on the effective creep properties will be presented, building on the simpler grain-scale models of Takei & Holtzmann [26].

## Figures and Tables

**Figure 20.**
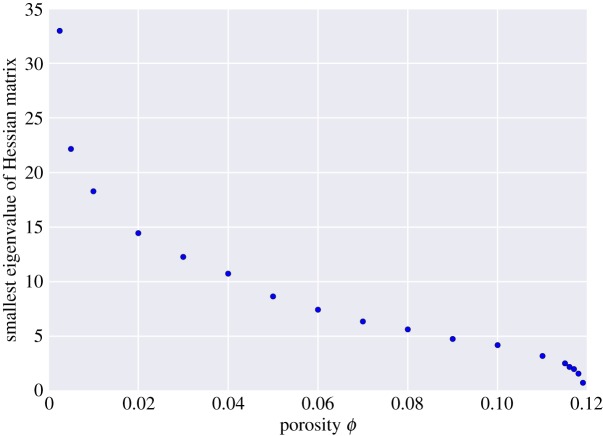
Smallest eigenvalue of the Hessian matrix for ‘c’ topologies and *θ*=40^°^. The topology becomes unstable as the porosity approaches 12%.

## Data accessibility

Surface Evolver scripts for generating the melt topologies are available online at www.johnrudge.com/melt, along with data tables of energy, pressure, contact areas and permeabilities.

## Appendix A. Numerical methods

**(a) Problem definition**

The variational problem of minimizing *J* in (2.2) is discretized by representing the solid–liquid interface by a triangulation as shown in figure 2. Owing to the imposed symmetry, points on the boundary of the computational domain are constrained to lie on certain symmetry planes. In figure 2*a*, there are five such planes. Two of these planes are associated with the triple lines where two solid–liquid surfaces meet a solid–solid surface, one corresponding to a square face (left), the other to a hexagonal face (right). Two more of the symmetry planes are where the melt junction is truncated in the figure, marking the middle of one of the grain edges. The final plane is an additional mirror plane (top left). Of the five symmetry planes, four are mirror planes, but one is not: the plane associated with the hexagonal solid–solid contacts. The melt surface on the opposite side of the hexagonal triple line is related by a 180^°^ rotation, not a reflection. This is in contrast to the problem of a melt junction with tetrahedral symmetry, where the fundamental computational domain is bounded solely by mirror planes. Care needs to be taken in constructing the mesh so that the edges on the hexagonal triple line respect the rotational symmetry. Fortunately, the Surface Evolver software provides functionality for such symmetry, allowing edge elements to ‘wrap’ under a symmetry operation (see [17] for further details).

All the quantities in *J* can be calculated by numerical approximations of the appropriate integral quantities. The simplest of these is the total area of solid–liquid contact, which is the surface integral,

A 1 $$A_{\mathrm{sl}}=\int_{S_{\mathrm{sl}}}dS,$$

and when discretized simply involves summing the areas of the individual triangles. The solid–solid surface is not triangulated, but its area can be calculated from knowledge of its bounding curve (the triple line) because of the assumption that solid–solid surfaces are flat. The area is given by an application of Stokes’ theorem as

A 2 $$A_{\mathrm{ss}}=\frac{1}{2}\int_{\Gamma_{t}}\text{n}\times (\text{x}-\text{p})\cdot d\text{x},$$

where **p** is the position vector of the point at the centre of a given solid–solid contact, **x** is the position vector of a point on the triple line, **n** is a vector normal to the solid–solid interface and *Γ*_*t*_ is the triple line curve. The volume of liquid can be written as a surface integral using the divergence theorem,

A 3 $$V_{l}=\frac{1}{3}\int_{S}\text{x}\cdot \text{n}\;dS,$$

where the closed surface *S* encloses the liquid and **n** is the outward normal from the liquid. Part of the surface *S* is the solid–liquid interface; the rest consists of parts of the planes which bound the computational domain due to the symmetry. As these are planes, **x**⋅**n** is constant over these parts of the surface *S*, and the surface integral can be further reduced to a line integral similar to (A.2). Furthermore, coordinates are chosen so that the centre of the melt junction is the origin of the coordinates. As three of the planes (all but the two associated with the truncation) go through the origin, **x**⋅**n**=0, and there is no contribution to the surface integral from these planes.

**(b) Optimization**

The Surface Evolver script that solves for the equilibrium geometries proceeds by a series of optimization and refinement steps. The initial mesh is very coarse, consisting of four triangles that represent a simple planar surface with vertices lying on the five bounding planes. The mesh is then alternately optimized, refined and ‘groomed’. Initial stages of optimization are performed using gradient decent; later stages are performed using Newton’s method. The mesh is continually refined until individual mesh edges are 0.025 times the grain edge length. ‘Grooming’ swaps mesh edges so that triangles are roughly equiangular. The eigenvalues of the Hessian matrix are checked to ensure all solutions have converged to a minimum (with all eigenvalues positive), not a saddle point, of the energy.

**(c) Different topologies**

The different topologies explored in this contribution have different problem set-ups, essentially due to the removal of one or more of the bounding planes from the connected ‘c’ topology problem. For example, to find ‘s’ solutions that wet the square faces, the bounding plane corresponding to the square triple line was removed. To find isolated ‘i’ solutions, the two planes associated with truncation were removed. To find hex-wetted ‘h’ solutions, the plane associated with the hexagon triple line was removed.

**(d) Accuracy of solutions**

The accuracy of the numerical solutions has been checked by comparing them against analytical solutions for combinations of parameters for which such solutions exist (see appendix B). For example, for an ‘s’ type solution with a dihedral angle of 60^°^ that consists of spherical surfaces, the numerical mean curvature differs from the analytical expression (B.11) by only 0.004%. As the spacing between mesh edges has been kept fixed, the solutions at low porosity are liable to be less accurate than those at higher porosity as fewer mesh nodes are used. However, the good comparison with the expected behaviour at low porosity suggests they are well resolved.

**(e) Regime diagram**

To generate the regime diagram depicted in figure 4, a series of simulations were run for different parameter combinations in intervals of 1% for porosity and 5^°^ for dihedral angle. The boundaries between different topologies were determined by two different methods depending on the nature of the boundary. The first type of boundary occurs when the topology assumed in the calculation is inconsistent with the tessellation. For example, when calculating the isolated topologies, there is a point at which the individual isolated pockets of melt meet as porosity increases. This can be determined by finding the porosity at which the extent of the isolated pocket is such that it extends to the middle of a grain edge. Similarly, the lower boundary of the ‘s’ topology can be determined by when the grain–melt surface near the square face first touches the boundary of the unit cell. This type of boundary is straightforward to determine accurately, and can be obtained through a simple interpolation of results at different porosities.

The second type of boundary is more difficult to constrain. This occurs when the topology in question becomes unstable as the parameters change. An example of this type of boundary is where ‘c’ topologies no longer exist. This type of boundary can be identified by the lowest eigenvalue of the Hessian matrix [16], as exemplified by figure 20. As porosity increases, the lowest eigenvalue decreases until at some point it reaches zero and the surface is no longer a minimum of the energy, but a saddle point. Following [16], the boundaries of such topologies have been estimated based on linearly extrapolating to the point at which the lowest eigenvalue reaches zero. As the method is based on extrapolation, it is inherently somewhat inaccurate. Additional simulations have been run in the vicinity of boundaries to improve the accuracy (see additional points near 12% in figure 20). Such boundaries are expected to be accurate to within at least ±0.5% and ±5^°^.

## Appendix B. Analytical and approximate solutions

**(a) Spherical surfaces**

In certain special cases, the melt geometry can be calculated analytically, and these cases form useful tests of the numerics. These analytical cases arise when the solid–liquid interface is spherical. The first of these cases is for the disaggregated topologies ‘d’, where the grains are isolated spheres. The energy and curvature are then given by

B 1 $$E=\gamma_{\mathrm{sl}}d^{2}\pi^{1/3}(3{\left(1-\phi )\right)}^{2/3}$$

and

B 2 $$H=\frac{1}{d}{\left(\frac{8\pi }{3(1-\phi )}\right)}^{1/3}.$$

The second case arises for the ‘i’ topologies, when the dihedral angle is *θ*=180^°^ and the melt forms spherical bubbles at the grain corners. Quantities of interest in this case are

B 3 $$\begin{array}{rl} A_{\mathrm{sl}} & =24\pi d^{2}{\left(\frac{\phi }{16\pi }\right)}^{2/3}, \end{array}$$

B 4 $$\begin{array}{rl} A_{\mathrm{ss}} & =\frac{3}{4}(1+2\sqrt{3})d^{2}-22\pi d^{2}{\left(\frac{\phi }{16\pi }\right)}^{2/3}, \end{array}$$

B 5 $$\begin{array}{rl} E & =\gamma_{\mathrm{sl}}A_{\mathrm{sl}} \end{array}$$

B 6 $$\begin{array}{rl} \mathrm{and}\;H & =-\frac{1}{d}{\left(\frac{16\pi }{\phi }\right)}^{1/3}. \end{array}$$

Finally, the square-wetted topologies ‘s’ are also spherical for particular choices of porosity and dihedral angle (see (B.10) below). The grain takes the shape of a sphere with eight spherical caps removed (corresponding to the hexagonal faces of the unit cell). Quantities of interest are given by

B 7 $$\begin{array}{rl} \phi & =1+\frac{\sqrt{3}\pi }{16}\left(6-9\cos\frac{\theta }{2}+\cos\frac{3\theta }{2}\right)\sec^{3}\frac{\theta }{2}, \end{array}$$

B 8 $$\begin{array}{rl} H & =\frac{4\cos(\theta /2)}{d\sqrt{3}}, \end{array}$$

B 9 $$\begin{array}{rl} A_{\mathrm{sl}} & =\frac{3\pi }{4}d^{2}\sec\frac{\theta }{2}\left(4-3\sec\frac{\theta }{2}\right) \end{array}$$

B 10 $$\begin{array}{rl} \text{and}\;A_{\mathrm{ss}} & =\frac{3\pi }{2}d^{2}\tan^{2}\frac{\theta }{2}. \end{array}$$

These solutions exist from a dihedral angle of zero (where the porosity is at the disaggregation value, $\phi =1-\pi \sqrt{3}/8\approx 32\%$) to a dihedral angle of 60^°^, where $\phi =1+\pi -\pi 3\sqrt{3}/4\approx 6\%$, which lies at the boundary between ‘c+s’ and ‘c’ in figure 4.

**(b) Tube approximation**

In addition to the analytical solutions provided above, there are some approximate solutions that are useful for providing insight into the behaviour at small porosities. One such approximate solution assumes that the melt resides along tubes of uniform cross section along the grain edges (e.g. [21,29]). The channel cross-sectional area is related to the porosity by

B 11 $$A_{\mathrm{ch}}\approx \frac{\sqrt{2}}{12d^{2}\phi }.$$

For small dihedral angles, a good approximation to the cross-sectional shape of the channel is given by the region between three touching identical circles. In this case, the radius *r* of the circles is related to the channel area by

B 12 $$A_{\mathrm{ch}}=r^{2}\left(\sqrt{3}-\frac{\pi }{2}\right).$$

Combining (B.14) and (B.15) yields the following expression for the mean curvature:

B 13 $$H=\frac{1}{d}\sqrt{\frac{3(\sqrt{3}-\pi /2)}{\sqrt{2}}}\phi^{-1/2}.$$

Assuming *γ*_*ss*_≈2*γ*_*sl*_ (appropriate for *θ*≈0), other quantities of interest can be written as

B 14 $$\begin{array}{rl} A_{\mathrm{ss}} & =A_{\mathrm{cell}}-3\sqrt{\frac{2\sqrt{2}}{\left(\sqrt{3}-\pi /2\right)}}d^{2}\phi^{1/2}, \end{array}$$

B 15 $$\begin{array}{rl} A_{\mathrm{sl}} & =\pi \sqrt{\frac{3\sqrt{2}}{2(\sqrt{3}-\pi /2)}}d^{2}\phi^{1/2} \end{array}$$

B 16 $$\begin{array}{rl} \text{and}\;E & =\gamma_{\mathrm{sl}}A_{\mathrm{cell}}\left(1-\frac{4}{3}\frac{\sqrt{6\sqrt{2}\left(\sqrt{3}-\pi /2\right)}}{1+2\sqrt{3}}\phi^{1/2}\right). \end{array}$$

## Appendix C. Calculation of permeability

The effective permeability of the melt networks was calculated by solving the appropriate Stokes flow problem for the unit cell, and then upscaling using the results from periodic homogenization theory (e.g. [28,35]). In dimensionless form, the cell problem to solve is

C 1 $$\nabla^{2}{\text{u}}_{j}=\nabla P_{j}-{\text{e}}_{j}$$

and

C 2 $$\nabla \cdot {\text{u}}_{j}=0$$

for velocities **u**_*j*_ and pressures *P*_*j*_; **e**_*j*_ is the *j*th unit vector (*j*=1,2,3). No-slip (**u**_*j*_=**0**) boundary conditions are applied to the solid–liquid boundaries, and appropriate periodic boundary conditions are applied on the boundaries of the unit cell. The permeability tensor is then given by

C 3 $$k_{ij}=\frac{1}{V}\int u_{ji}\;dV,$$

where *u*_*ji*_ is the *i*th component of velocity **u**_*j*_ and *V* is the volume of the unit cell. Owing to the cubic symmetry, the permeability tensor must be isotropic, and hence it suffices to solve the Stokes flow problem only for *j*=1. A tetrahedral mesh of the liquid domain for the unit cell was generated with mesh edge size equal to that used for the triangulation of the solid–liquid surface. Equations (C.20) and (C.21) were solved numerically using Taylor–Hood finite elements (second-order velocity, first-order pressure) with the FEniCS software [36,37].
